# Two-session Radiosurgery as Initial Treatment for Newly Diagnosed Large, Symptomatic Brain Metastases from Breast and Lung Histology

**DOI:** 10.7759/cureus.5472

**Published:** 2019-08-24

**Authors:** Eduardo E Lovo, Leonel B Torres, Fidel J Campos, Victor E Caceros, Kaory E Barahona, Mario H Minervini, William A Reyes

**Affiliations:** 1 Radiosurgery, International Cancer Center, Diagnostic Hospital, San Salvador, SLV; 2 Nerosurgery, International Cancer Center, Diagnostic Hospital, San Salvador, SLV; 3 Radiation Oncology, International Cancer Center, Diagnostic Hospital, San Salvador, SLV

**Keywords:** brain metastases, radio-surgery, fractionated radio surgery, neuro-oncology, brain surgery

## Abstract

Introduction

Surgery is considered the treatment of choice for patients with large, symptomatic brain metastases. This report describes a series of patients treated with upfront two-session radiosurgery rather than surgery for large brain metastases from breast and lung histology.

Methods

From October 2016 to January 2019, 10 consecutive patients with neurologic symptoms from large brain metastases producing mass effects underwent two sessions of radiosurgical treatments 30 days apart. The response was assessed by imaging and clinical evaluations.

Results

Ten patients had a total of 36 tumors; of these, 22 lesions with a mean volume of 12.3 ml (range, 7-78.4 ml) underwent two-session radiosurgery. The mean prescription dose for the first treatment was 13 Gy (range, 9-18 Gy) to the 50% isodose line, and the intratumoral mean dose was 17.9 Gy (12-22.9). All 10 patients had neurological symptoms, with a mean Karnofsky physical score (KPS) of 60 (range, 50-70) on the day of treatment. None of these patients required neurosurgical or emergency consultation related to worsening of neurological symptoms between the first and second treatments. At 30 days, the mean KPS was 80 and maintained at 80 at the last follow-up (range, 60-100; P=0.002), and mean lesion volume was 4.1 ml (range, 1.3-70 ml). The mean prescription dose for the second treatment was 12 Gy (range, 9-18 Gy) to the 50% isodose line, and the intratumoral mean dose was 17.9 Gy (11-22.4). The mean overall survival was 24 months (range, 3-32 months). At last follow-up, three patients (30%) had died, two of systemic progression and one of tumor progression, and at one year, local tumor control was 91% and 19 (86%) lesions showed documented local control at last follow up. In those tumors that progressed, the mean time to progression was eight months (range, 5-20 months), and the mean time to surgery was nine months (range, 5-32 months).

Conclusion

Two-session radiosurgery proved to be a safe treatment for patients with large, symptomatic metastases in this series. Neurological worsening after radiosurgery for large lesions of breast and lung histology may be an infrequent event. This strategy in radiosurgery may have neurological benefits for these patients providing adequate local tumor control while reducing the need of upfront surgery at diagnosis.

## Introduction

Brain metastases are the most common type of intracranial tumors, with incidence rates of eight to 14 per 100,000 persons [1-2]. The development of targeted systemic, as well as focal therapies, has improved extracranial disease control and increased the long-term survival of patients with brain metastases while minimizing risks and adverse effects. The treatment of brain metastases requires a multimodal approach, usually consisting of open or minimally invasive surgery, radiation, and chemotherapy or immunotherapy [3-6].

The 2019 National Comprehensive Cancer Network (NCCN) guidelines, V1, recommend that surgery for patients with limited brain metastases, whether newly diagnosed or with stable systemic disease, be considered to manage mass effect or neurological symptoms, usually produced by tumors >2 cm. According to the NCCN, the evidence-based guidelines of the Congress of Neurological Surgeons recommend surgery in adults with metastatic brain tumors if the brain metastases are large, have significant perilesional edema, result in neurological deficits, and present with uncertain pathology [7].

Although surgery as the initial treatment is effective in patients with newly diagnosed large brain metastases, it nevertheless carries intrinsic risks for patients [8]. These drawbacks can include further neurological deficits; wound complications; leptomeningeal spreading of the disease, especially in the posterior fossa; residual tumor associated with reduced survival; and possible delay in further oncological therapies that are needed for systemic control [9-11].

Shaw et al. recommend that radiosurgery doses to large lesions be limited, with reference doses of 24 Gy recommended for lesions <2 cm in diameter, doses of 18 Gy recommended for lesions >2 cm and <3 cm in diameter, and doses of 15 Gy recommended for lesions >3 cm [12]. Nevertheless, doses below 20 Gy were found insufficient to provide long-term (i.e., one-year) control by several authors [13-16].

Various radiosurgical schemes have been devised to deliver higher radiation doses through different fractionation strategies. Three-stage and two-stage radiosurgeries were devised for the treatment of large metastases, mostly in patients requiring placement of an invasive frame with Gamma Knife®. This treatment consists of the initial delivery of a relatively large dose of 15 Gy or 10 Gy depending on lesion volume and size, followed by doses at 15 and/or 30 days to a total dose of 30 Gy [17-20]. The present study describes our initial experience with first-line two-stage radiosurgery rather than surgery in patients newly diagnosed with large symptomatic brain metastases (>7.5 ml) using a stereotactic rotating gamma-ray system.

## Materials and methods

The present study is a retrospective series of 10 consecutive cancer patients who were newly diagnosed with large metastatic brain lesions suspected of causing neurological symptoms between October 2016 and January 2019 and referred for surgical treatment. This study was approved by the ethics committee of our institution, and all patients provided informed consent for treatment. All patients were evaluated by a team of neurosurgeons and deemed operable, as none of the large lesions suspected of causing neurological symptoms were in deep structures, such as the basal ganglia or brainstem and that despite the presence of comorbidities they could be safely operated under awake craniotomy technique or general anesthesia. All patients were advised that surgery was recommended as the treatment of choice in accordance with NCCN guidelines and current standards, with those wishing to avoid surgery were advised to undergo two-session radiosurgery. All patients were informed that if their neurological symptoms worsened or did not improve after radiosurgery, immediate surgery would be recommended. Images were taken during the next treatment at 30 days and every three months to evaluate response or progression.

Two-session radiosurgery technique

**Figure 1:**
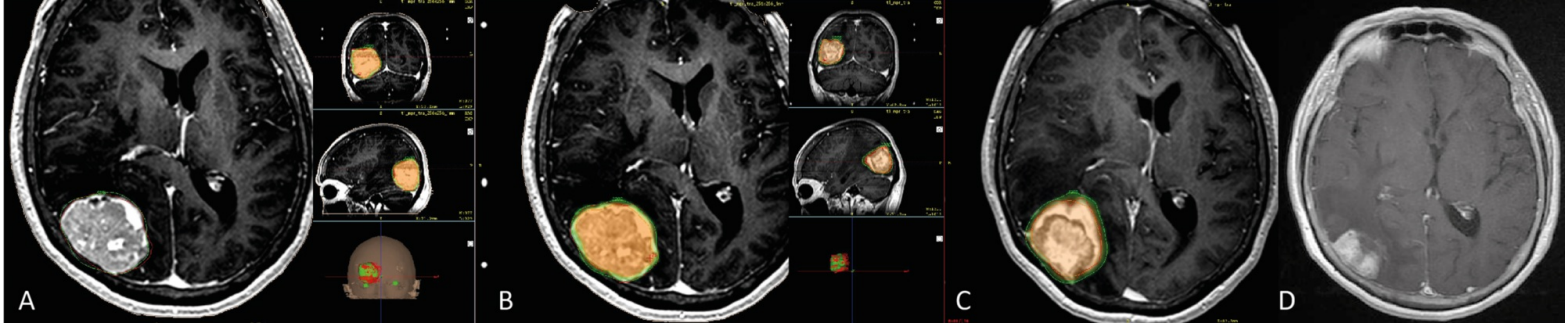
Two-session radiosurgery to a single occipital metastasis A. T1 gadolinium axial image of a 49-ml lesion with breast histology on the right occipital lobe associated with a mass effect. B. Radiation dose during the first session of radiosurgery was 12 Gy to the 50% isodose line, with the orange area representing everything inside the tumor receiving a dose of 15–24 Gy. C. Thirty days after the first radiosurgery session, the lesion volume was 33 ml (32% reduction). The patient was treated with a second session of radiosurgery, consisting of a dose of 12 Gy to the 50% isodose line, with the orange area representing everything inside the tumor receiving a dose of 15–24 Gy. D. After five months, the tumor volume was 22 ml (55% reduction).

**Figure 2:**
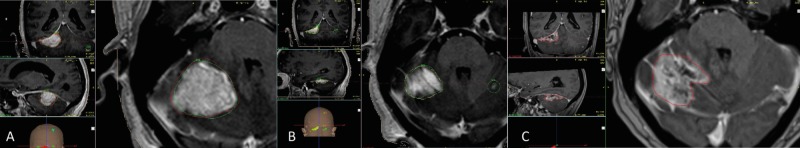
Two-session in a large posterior fossa metastases in a multiple lesion case A. Three-dimensional T1 gadolinium scan showing a large metastasis (11.5 ml) with breast histology in the posterior fossa with a mass effect over the fourth ventricle. Radiation dose during the first session of radiosurgery was 15 Gy to the 50% isodose line. B. Thirty days after the first radiosurgery session, the lesion volume was 4.5 ml (60% reduction), the patient underwent a second session of radiosurgery, during which the dose was 15 Gy to the 50% isodose line. C. Eight months after the initial treatment, the lesion remains controlled.

**Figure 3:**
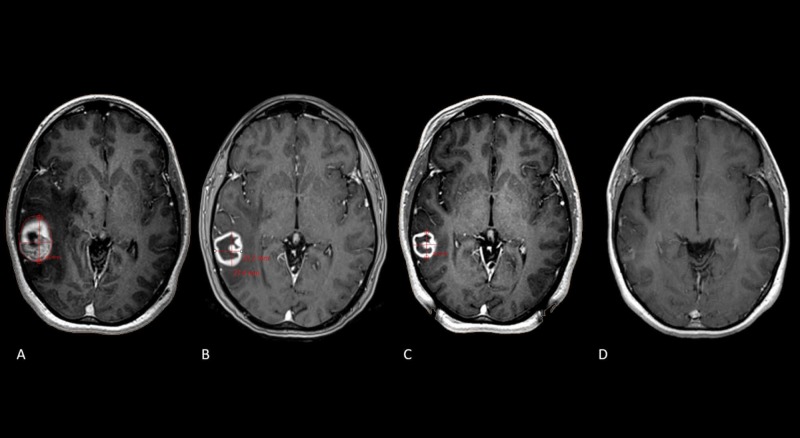
Two-session radiosurgery for a solitary right temporal metastasis Sequence of images in time. A. Axial T1 gadolinium showing a single right temporal brain metastasis producing mass effect from a primary breast histology with hormone negative receptors. Initial volume at the day of treatment was 15.9 ml; this lesion received 15 Gy to the 50% isodose line. B. Axial T1 gadolinium at 10 days after the first radiosurgery showing a 53% reduction of the original tumor volume, it them measured 7.5 ml with a resolution of the mass effect over the midline. C. Axial T1 gadolinium at the second treatment at 30 days, the lesion now measures 4 ml with a reduction of 76% of the original tumor volume. D. Axial T1 gadolinium at 18 months after the initial treatment showing a complete tumor response.

Most patients underwent radiosurgery on an outpatient basis, with 8-mg doses of dexamethasone administered intravenously on the day of treatment, followed by 2-mg doses of oral dexamethasone every eight hours for one week or as needed until the patient was clinically improved and stable. Patients fasted six hours on the day of the procedure and were administered local anesthetic for stereotactic frame placement provided by Infini™ (Masep Medical Company, Shenzhen, China) the procedure was done by a neurosurgeon, patients underwent magnetic resonance imaging (MRI) with a 1.5-tesla Avanto™ (Siemens Corporation. Erlangen, Germany), usually consisting of only one volumetric T1 delayed (usually 15 minutes before MRI), double dose contrast of 1.0 to 1.5-mm slice thickness with no spacing of the head (apex to foramen magnum). Images were transferred to the treatment planning station (Superplan®). Organs at risk, including the visual pathway and brainstem, were contoured by neurosurgery if needed, and the planned target volume was usually contoured by radiation therapy specialists and neurosurgeons. All visible lesions were irradiated, with those <2 cm in diameter receiving single doses of ≥21 Gy. Lesions 2 cm to 3 cm, 3 cm to 4 cm, and >4 cm in diameter received first treatments of 18 Gy, 15 Gy, and 12 Gy, respectively, typically to the 50% isodose line and scheduled for subsequent treatments based on a two-session radiosurgery protocol. Patients were evaluated 48 hours and one week after initial treatment. Thirty days after the initial treatment, patients were reevaluated neurologically for symptoms and proceeded with the second session of radiosurgery. Stereotactic MRI acquisition was repeated as described earlier, and replanning was based on the new volume of the lesion (Figures 1-3).

These patients were treated such that the total prescribed dose of the first and second treatments was as close to 30 Gy as possible. If new small lesions were detected at 30 days, they were also treated.

## Results

Of the 10 patients, seven had primary breast cancer (70%) and three had lung cancer (30%). Seven patients (70%) had multiple metastases, and 30% had single lesions. These 10 patients had a total of 36 tumors; of these, 22 (mean volume, 12.3 ml; range, 7-78.4 ml) were subjected to two-session radiosurgery protocol. Twelve tumors (54%) were located supratentorially, and 10 (46%) were located infratentorially. The mean dose prescribed to the 50% isodose line was 13 Gy (range, 9-18 Gy), the mean percentage of target coverage was 94% (range, 92-100%) with a 0.81 conformality, and the average treatment time was 64.8 minutes (range, 26-209 minutes) and intratumoral mean dose was 17.9 Gy (12-22.9). All 10 patients had neurological symptoms, with a mean Karnofsky physical score (KPS) of 60 (range, 50-70) on the day of initial treatment. The most frequent symptoms were a headache (80%), hemiparesis (50%), visual disturbances (40%), gait imbalance and ataxia (40%), and dysphasia (30%). Subjective neurological improvement was observed in seven patients 48 hours after treatment in all 10 patients one week after treatment time. None of the patients required neurosurgical or emergency consultation related to worsening of neurological symptoms between the first and second treatments. At the second treatment, 30 days after the first treatment, the mean KPS was 80 and was maintained at 80 at last follow-up (range, 60-100; *P *= 0.002); of the patients alive at last follow-up, one had neurological dysfunction attributable to tumor progression and cerebrospinal fluid dissemination or treatment effects (Table 1).

**Table 1:** Patient characteristics KPS, Karnofsky physical score; CNS, central nervous system; HR, hormone receptor; ND, neurological dysfunction; SCLC, small cell lung carcinoma

N.	Age	Sex	Histology	Subtype*	Single lesion	Pre KPS	Tumor Vol**	KPS at 30 days	Tumor vol at 30 days	KPS at last Follow-up	ND***	Surgery	Distal progression	Survival	Alive	Died of CNS progression
1	77	Fem	Breast	HR+	Yes	50	9.8	80	2.2	80	No	Yes	No	33	Yes	No
2	37	Fem	Breast	HR-	Yes	50	17.2	100	4.3	80	No	No	No	20	Yes	No
3	64	Male	Lung	SCLC	No	40	18.7	60	1.3	60	Yes	Yes	Yes	26	Yes	No
4	68	Fem	Breast	HR+	No	60	78.4	70	70	0	No	No	No	6	No	Yes
5	69	Male	Lung	SCLC	Yes	50	11.7	80	2.5	0	No	No	No	3	No	No
6	42	Fem	Breast	HR+	No	60	11.5	90	4.9	90	No	No	No	13	Yes	No
7	60	Fem	Breast	HR+	No	60	23.8	80	8.5	0	No	No	Yes	12	Yes	No
8	60	Fem	Lung	SCLC	No	60	7.6	80	2.3	80	No	No	Yes	13	Yes	No
9	56	Fem	Breast	HR+	No	50	14	70	5.5	80	No	No	Yes	10	Yes	No
10	67	Fem	Breast	HR-	Yes	80	49.6	100	33.2	90	No	No	No	5	Yes	No
* HR+ at least one or both hormone receptors present, HR both hormone receptors absent,																
** Tumor volume of the largest lesion to receive adaptive radiosurgery and suspected to be causing neurological symptoms.																
***Neurological dysfunction at last follow-up																

The mean lesion volume was 4.1 ml (range, 1.3-70 ml), equal to a 66% reduction. Of the 22 lesions, 21 (95.4%) showed substantial responses of 11.7 and 3.9 ml after the first and second treatments, respectively. The remaining lesion, which was cystic, experienced a modest reduction in volume of 11%. The mean dose for the second adaptive treatment to the 50% isodose line was 12 Gy (range, 9-18 Gy), the mean percentage of target coverage was 96% (range, 94-100%) with a 0.81 conformality, and the mean treatment time was 35.4 minutes (range, 13.5-77 minutes) and the intratumoral mean dose was 17.9 Gy (11-22.4).

**Figure 4:**
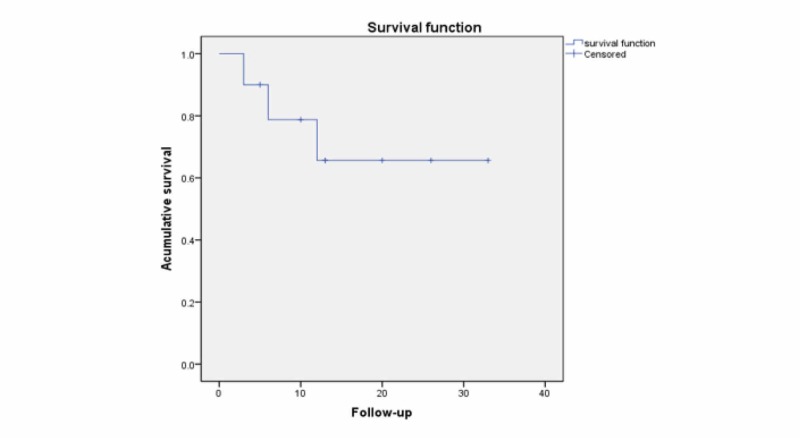
Mean overall survival curve

The mean overall survival was 24 months (range, 3-32 months), 69% were alive at one year with a local tumor control of 91% (Figure 4).

**Figure 5:**
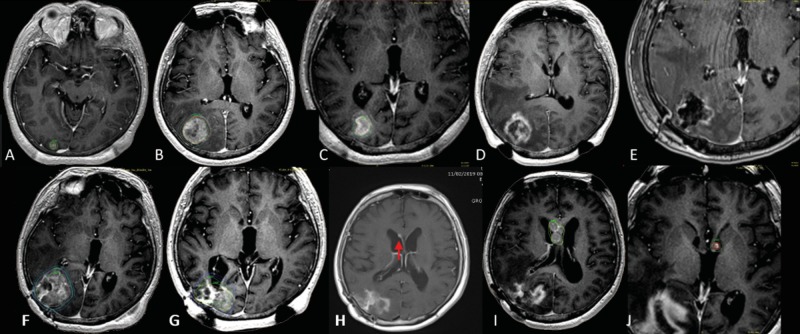
Two-session radiosurgery followed by surgery in a multiple metastases case in a lung cancer patient A. Initial plan showing a small occipital metastasis being treated with a single dose of 20 Gy to the 65% isodose line. B. Lesion progression eight months after initial treatment. Due to the size and volume (18.7 ml) of the lesion, the patient received a second course of two-session radiosurgery, at a dose of 15 Gy to the 46% isodose line. C. Thirty days later, the lesion volume was 1.3 ml, and the patient received a second session and third course of radiosurgery, at a dose of 11 Gy to the 50% isodose line. D Eight months later, 16 months after the initial treatment, the tumor had progressed, and the patient underwent surgery. E. Three-dimensional MRI after surgery, showing an apparent residual in the most medial aspect of the cavity; further radiation treatment was not prescribed. F. Four months after surgery, local tumor progression was observed, with the tumor volume being 38.2 ml, and the patient was administered a new course of two-session radiosurgery, consisting of a dose of 12 Gy to the 50% isodose line. G After 30 days, tumor response was adequate, with a tumor volume of 14.4 ml (62% reduction). The patient was administered the second session of radiosurgery, consisting of a dose of 12 Gy to the 50% isodose line. H. Eight months after the second course of two-session radiosurgery and 24 months after initial treatment, the occipital lesion appeared controlled, but a small intraventricular metastasis was observed (red arrow). I and J. Results of rescue radiosurgery at nine months, 25 months after initial treatment for intraventricular tumor dissemination.

Three patients (30%) died, two of systemic progression, and one of local tumor progression. At last follow-up, 19 (86%) of the 22 lesions showed local control, two tumors (9%) experienced complete response, 13 (59%) had a partial response, three (13.6%) has stable disease and four (18.1%) experienced tumor progression. In those tumors that progressed, the mean time to progression was eight months (range, 5-20 months), and the mean time to surgery was nine months (range, 5-32 months). One patient required surgery due to tumor progression eight months after treatment. Eight months after surgery, this patient showed evidence of leptomeningeal dissemination to the ventricles, was stabilized by radiosurgery, and remained alive at last follow-up (Figure 5).

**Figure 6:**
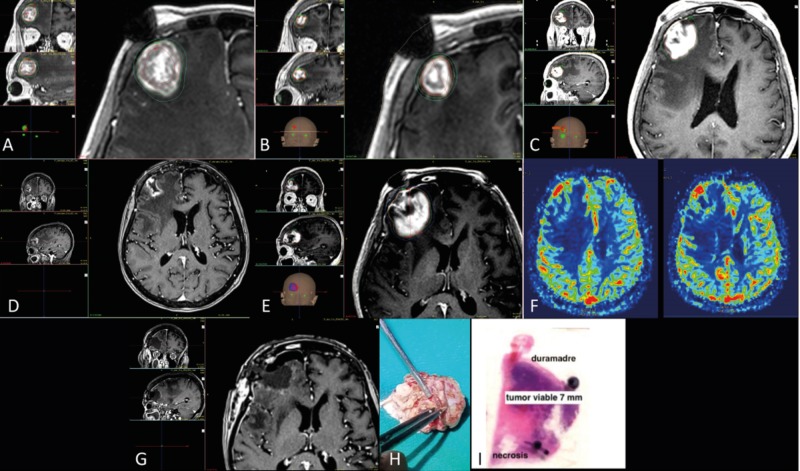
Two-session, rescue radiosurgery and surgery in a single brain metastasis A. Three-dimensional T1 gadolinium scan showing a right frontal lesion with breast histology associated with mass effect. Radiation dose during the first session of radiotherapy was 18 Gy to the 50% isodose line. B. Thirty days later, the patient underwent a second session of radiosurgery, during which the dose was 15 Gy to the 50% isodose line. C. Following disease progression at 20 months, surgery was recommended, but the patient refused and underwent a third course of radiosurgery, during which the dose was 15 Gy to the 50% isodose line. D. Adequate tumor response 30 months after the original treatment and 10 months after the third radiosurgery session. E. Tumor progression and radionecrosis at 33 months. F. Perfusion MRI predicting viable tumor only in the part of the lesion next to the dura mater. G. Postoperative MRI showing complete removal of the lesion one week after neoadjuvant radiosurgery with 15 Gy to the 50% isodose line. H. En-bloc tumor resection, including removal of the transition zone between the tumor and the dura mater, which was also removed. I. Histologic confirmation that viable tumor (tumor viable) 7 mm in size was present only in the portion in contact with the dura mater (duramadre), whereas the rest was necrotic (necrosis), as predicted by perfusion MRI.

A second patient, with documented progression at 20 months, was managed with radiosurgery and remained stable until progressing at 32 months and underwent surgery (Figure 6).

A third patient died of tumor progression five months after the second treatment, with neurosurgical treatment not available at the treating hospital. Two patients (20%) demonstrated distal progression and were treated by second- and third-course radiosurgery. Total documented local and distal progression at last follow-up was 50%.

## Discussion

Upfront surgery has been the treatment of choice for patients with large, symptomatic metastases regardless of the histology subtype. The classical rationale for surgery includes the prompt alleviation of mass effects and improvements in neurological deficits. In contrast, radiation and focal treatments such as radiosurgery were thought to function in a delayed fashion or be ineffective [7]. Administration of these treatments to large, mass effect-causing lesions was thought to be potentially dangerous, as these treatments may cause additional edema. None of the patients in our series experienced any form of neurological deterioration after radiosurgery; rather, neurological improvements were usually observed after treatments. The potential worsening thought to occur after radiosurgery for large lesions may, therefore, be unsupported by practical evidence, suggesting the need for further investigation.

Treatments in the current clinical series were based on our experience treating more than 24 patients with large tumors using a linear accelerator (LINAC) since 2011, using habitual dosing recommendations from Shaw et al.’s trial [12]. LINAC involved fractionated stereotactic radiation therapy, single-session SRS to large lesions plus whole-brain radiation therapy, and, after 2014, single session gamma SRS for newly diagnosed large symptomatic lesions, with doses based on lesion size. Due to technical limitations of our LINAC system, more traditional multifraction (i.e., three consecutive sessions of 9 Gy) were deemed relatively inferior compared to two-session radiosurgery with Infini (GammaRay) that could provide a steeper dose fall-off and typical higher intratumoral mean dose at an equal prescription dose [17-21]. In Shaw’s report, the multivariate analysis demonstrated that patients treated on a linear accelerator (versus the Gamma Knife) had a 2.84 greater risk of local progression at the same prescribed dose [12]. This difference in local progression could be explained by a higher intratumoral dose provided by Gamma systems. As our main goal was to avoid surgery in symptomatic patients, we felt that Infini would be more suitable for obtaining a quicker tumor response that hopefully traduced in faster neurological improvement. Further studies are needed to compare multifraction LINAC versus multifraction Gamma Knife and the results provided by two- or three-session Gamma Knife in the treatment of large brain metastases [18-20,22,23].

Our previous study analyzing the safety and tolerability of over 210 awake craniotomies for intrinsic brain lesions, including metastases, showed that complete resection rates were lower when tumors were localized in or near eloquent areas, and the rates of transient and permanent neurological worsening were higher as expected. Most importantly our group demonstrated that awake craniotomy technique avoids postoperative mechanical ventilation or cardiopulmonary complications in the majority of patients and is well tolerated even in those patients with comorbidities [24]. There were no medical contraindications that could not be overpassed to operating the patients described in this series apart from the hypothesis of this investigation that stated that surgery could be spared in the majority of patients with large symptomatic brain metastases as was demonstrated. Our version of neoadjuvant (presurgical) radiosurgery protocol was started in 2015 with the intention of reducing leptomeningeal seeding or the impact of tumor residuals, defining a more adequate target, and reducing radiation necrosis. Nevertheless, this protocol was quickly discontinued as an upfront treatment as patients neurologically recovered quickly following radiosurgery and steroid administration, suggesting that subsequent surgery may be unnecessary. Not surprisingly, metastasis size does not necessarily correlate with neurological symptoms. Rather, neurological symptoms tend to improve in patients administered steroids alone. Radiosurgery apparently enhances the effects of low-dose steroids (e.g., 0.5 mg dexamethasone), resulting in rapid patient improvement. Our neoadjuvant radiosurgery protocol was replaced by the present study of two-session radiosurgery, inspired by staged radiosurgery schemes described by others, but with the hope to benefit not only those unsuitable for surgery, but rather those who could be operated by current recommendations standards [22-23]. It was exactly this group of patients for whom we sought to provide an apparent non-inferior, safe, effective, noninvasive alternative means of radiosurgery for rapid tumor response and control. More importantly, we sought to relieve them of their neurological symptoms with outcomes comparable to those of surgical treatment.

Although deemed relatively safe, surgery carries intrinsic risks including cerebrospinal fluid (CSF) dissemination of tumor cells and, as verified by others, tumor residuals reduce life expectancy [9-11,25]. At our center, surgery for large brain metastases is performed after adaptive radiosurgery has failed, or one week after radiosurgery if histology confirmation is needed. Surgical techniques include complete ultrasound-guided supramarginal en-bloc resection, including dura removal when there is evidence of tumor contact (Figure 5), especially for lesions in the posterior fossa, thereby hopefully limiting leptomeningeal spread and reducing the possibilities of tumor residuals. To briefly touch in histology, in our local practice, brain metastases from melanoma and renal cell carcinoma are very rare. These histology subtypes are considered more radioresistant than the histology here presented and thus may limit the extrapolation of the results here described.

Several recent studies have described different fractionation schemes that are intended to safely increase the biologically effective dose to large lesions, thereby improving long-term local control and survival when compared with historical reports of tumor control at one year of 45 and 49% in patients that received a lower dose in a single fraction such as 15 and 18 Gy, respectively [14,16-20,22-23]. Recent results suggest that the classical scheme of surgery followed by SRS may be comparable to staged (two-session) radiosurgery with local tumor failure rate at one year of 8% [26]. A prospective randomized trial concerning postoperative radiosurgery versus observation for completely resected brain metastases provide evidence of one-year local tumor failure rate of 28% for surgery followed by SRS versus 57% in the observation arm [27]. In a review of the literature of resection cavity radiosurgery for intracranial metastases, the one-year local control ranged between 74-91.5% [11]. Our series, although small, achieved a one-year local tumor control of 91%, and no patients required rescue surgery or experienced worsening of their symptoms during the period between radiosurgical treatments, with the mean time to rescue surgery after the second treatment being eight months. If surgery is to be undertaken due to tumor progression after two-session radiosurgery, we recommend our patients receive neoadjuvant radiosurgery before surgery.

As mentioned, leptomeningeal spread through surgery is a serious complication, one of our patients experienced distal disease progression through CSF dissemination of tumor cells, indicating that surgery, as executed in this case, was potentially detrimental. Larger series comparing first-line two-session or fractionated radiosurgery with surgery followed by SRS are needed to determine the best approach to patients with large, symptomatic metastases and selected histologic subtypes and adequate clinical settings.

## Conclusions

Two-session radiosurgery proved to be a safe treatment for patients suffering from large, symptomatic metastases from breast or lung histology in this series. The local failure rate at one year is comparable to other staged radiosurgery series and comparable to the most optimistic surgery followed by SRS series. Neurological worsening after radiosurgery for large lesions of theses histology may be an infrequent event. Further studies are needed to provide optimal timing and dosing recommendations.

## References

[1] Smedby KE, Brandt L, Backlund ML, Blomqvist P (2009). Brain metastases admissions in Sweden between 1987 and 2006. Br J Cancer.

[2] Walker AE, Robins M, Weinfeld FD (1985). Epidemiology of brain tumors: the national survey of intracranial neoplasms. Neurology.

[3] Alattar AA, Bartek J Jr, Chiang VL, Mohammadi AM, Barnett GH, Sloan A, Chen CC (2019). Stereotactic laser ablation as treatment for brain metastases recurring after stereotactic radiosurgery: a systematic literature review. World Neurosurg.

[4] Hong CS, Deng D, Vera A, Chiang VL (2019). Laser-interstitial thermal therapy compared to craniotomy for treatment of radiation necrosis or recurrent tumor in brain metastases failing radiosurgery. J Neurooncol.

[5] Lauko A, Thapa B, Venur VA, Ahluwalia MS (2018). Management of brain metastases in the new era of checkpoint inhibition. Curr Neurol Neurosci Rep.

[6] Shen CJ, Lim M, Kleinberg LR (2016). Controversies in the therapy of brain metastases: shifting paradigms in an era of effective systemic therapy and longer-term survivorship. Curr Treat Options Oncol.

[7] Nahed BV, Alvarez-Breckenridge C, Brastianos PK (2019). Congress of neurological surgeons systematic review and evidence-based guidelines on the role of surgery in the management of adults with metastatic brain tumors.. Neurosurgery.

[8] Pessina F, Navarria P, Cozzi L (2016). Role of surgical resection in patients with single large brain metastases: feasibility, morbidity, and local control evaluation. World Neurosurg.

[9] Patel KR, Burri SH, Asher AL (2016). Comparing preoperative with postoperative stereotactic radiosurgery for resectable brain metastases: a multi-institutional analysis. Neurosurgery.

[10] Munoz-Bendix C, Rapp M, Mijderwijk HJ (2019). Risk factors for in-brain local progression in elderly patients after resection of cerebral metastases. Sci Rep.

[11] Zhang Y, Chang EL (2014). Resection cavity radiosurgery for intracranial metastases: a review of the literature. J Radiosurg SBRT.

[12] Shaw E, Scott C, Souhami L, Dinapoli R, Kline R, Loeffler J, Farnan N (2000). Single dose radiosurgical treatment of recurrent previously irradiated primary brain tumors and brain metastases: final report of RTOG protocol 90-05. Int J Radiat Oncol Biol Phys.

[13] Amsbaugh M, Pan J, Yusuf MB (2016). Dose-volume response relationship for brain metastases treated with frameless single-fraction linear accelerator-based stereotactic radiosurgery. Cureus.

[14] Vogelbaum MA, Angelov L, Lee SY, Li L, Barnett GH, Suh JH (2006). Local control of brain metastases by stereotactic radiosurgery in relation to dose to the tumor margin. J Neurosurg.

[15] Varlotto JM, Flickinger JC, Niranjan A, Bhatnagar AK, Kondziolka D, Lunsford LD (2003). Analysis of tumor control and toxicity in patients who have survived at least one year after radiosurgery for brain metastases. Int J Radiat Oncol Biol Phys.

[16] Lehrer EJ, Peterson JL, Zaorsky NG (2019). Single versus multifraction stereotactic radiosurgery for large brain metastases: an international meta-analysis of 24 trials. Int J Radiat Oncol Biol Phys.

[17] Minniti G, Scaringi C, Paolini S (2016). Single-fraction versus multifraction (3 × 9 Gy) stereotactic radiosurgery for large (>2 cm) brain metastases: a comparative analysis of local control and risk of radiation-induced brain necrosis. Int J Radiat Oncol Biol Phys.

[18] Higuchi Y1, Serizawa T, Nagano O (2009). Three-staged stereotactic radiotherapy without whole brain irradiation for large metastatic brain tumors. Int J Radiat Oncol Biol Phys.

[19] Yomo S, Hayashi M, Nicholson C (2012). A prospective pilot study of two-session Gamma Knife surgery for large metastatic brain tumors. J Neurooncol.

[20] Yomo S, Hayashi M (2014). A minimally invasive treatment option for large metastatic brain tumors: long-term results of two-session Gamma Knife stereotactic radiosurgery. Radiat Oncol.

[21] Lovo E, Campos F, Caceros V, Minervini M, Reyes W (2018). Dosimetry and treatment descriptions using the first completely automated stereotactic intracranial radiosurgery rotating gamma ray unit in America. Cureus.

[22] Angelov L, Mohammadi AM, Bennett EE (2018). Impact of 2-staged stereotactic radiosurgery for treatment of brain metastases ≥ 2 cm. J Neurosurg.

[23] Dohm A, McTyre E, Okoukoni C (2018). Staged stereotactic radiosurgery for large brain metastases: local control and clinical outcomes of a one-two punch technique. Neurosurgery.

[24] Lovo E, Minervini M, Ahues E, Martinez-Cortez R, Milla R, Cruz C (2018). Safety and tolerability of awake craniotomy for brain tumors and other supratentorial lesions. Rev Argent Neuroc.

[25] Suki D, Abouassi H, Patel AJ, Sawaya R, Weinberg JS, Groves MD (2008). Comparative risk of leptomeningeal disease after resection or stereotactic radiosurgery for solid tumor metastasis to the posterior fossa. J Neurosurg.

[26] Dohm AE, Hughes R, Wheless W (2018). Surgical resection and postoperative radiosurgery versus staged radiosurgery for large brain metastases. J Neurooncol.

[27] Mahajan A, Ahmed S, McAleer MF (2017). Post-operative stereotactic radiosurgery versus observation for completely resected brain metastases: a single-centre, randomised, controlled, phase 3 trial. Lancet Oncol.

